# *Dnmt3b* regulates DUX4 expression in a tissue-dependent manner in transgenic D4Z4 mice

**DOI:** 10.1186/s13395-020-00247-0

**Published:** 2020-10-01

**Authors:** Linde F. Bouwman, Bianca den Hamer, Elwin P. Verveer, Lente J. S. Lerink, Yvonne D. Krom, Silvère M. van der Maarel, Jessica C. de Greef

**Affiliations:** 1 Department of Human Genetics, Leiden University Medical Center, Albinusdreef 2, 2333 ZA Leiden, The Netherlands; 2 Department of Neurology, Leiden University Medical Center, Albinusdreef 2, 2333 ZA Leiden, The Netherlands

**Keywords:** Facioscapulohumeral muscular dystrophy, DUX4, DNA methyltransferase 3B, D4Z4-2.5 mouse model, Mouse embryonic stem cells, Epigenetics, Lymphoid organs

## Abstract

**Background:**

Facioscapulohumeral muscular dystrophy (FSHD) is a skeletal muscle disorder that is caused by derepression of the transcription factor DUX4 in skeletal muscle cells. Apart from SMCHD1, DNMT3B was recently identified as a disease gene and disease modifier in FSHD. However, the exact role of DNMT3B at the D4Z4 repeat array remains unknown.

**Methods:**

To determine the role of Dnmt3b on DUX4 repression, hemizygous mice with a FSHD-sized D4Z4 repeat array (D4Z4-2.5 mice) were cross-bred with mice carrying an in-frame exon skipping mutation in *Dnmt3b* (Dnmt3b^MommeD14^ mice). Additionally, siRNA knockdowns of *Dnmt3b* were performed in mouse embryonic stem cells (mESCs) derived from the D4Z4-2.5 mouse model.

**Results:**

In mESCs derived from D4Z4-2.5 mice, Dnmt3b was enriched at the D4Z4 repeat array and DUX4 transcript levels were upregulated after a knockdown of *Dnmt3b*. In D4Z4-2.5/Dnmt3b^MommeD14^ mice, Dnmt3b protein levels were reduced; however, DUX4 RNA levels in skeletal muscles were not enhanced and no pathology was observed. Interestingly, D4Z4-2.5/Dnmt3b^MommeD14^ mice showed a loss of DNA methylation at the D4Z4 repeat array and significantly higher DUX4 transcript levels in secondary lymphoid organs. As these lymphoid organs seem to be more sensitive to epigenetic modifiers of the D4Z4 repeat array, different immune cell populations were quantified in the spleen and inguinal lymph nodes of D4Z4-2.5 mice crossed with Dnmt3b^MommeD14^ mice or Smchd1^MommeD1^ mice. Only in D4Z4-2.5/Smchd1^MommeD1^ mice the immune cell populations were disturbed.

**Conclusions:**

Our data demonstrates that loss of Dnmt3b results in derepression of DUX4 in lymphoid tissues and mESCs but not in myogenic cells of D4Z4-2.5/Dnmt3b^MommeD14^ mice. In addition, the Smchd1^MommeD1^ variant seems to have a more potent role in DUX4 derepression. Our studies suggest that the immune system is particularly but differentially sensitive to D4Z4 chromatin modifiers which may provide a molecular basis for the yet underexplored immune involvement in FSHD.

## Background

Facioscapulohumeral muscular dystrophy (FSHD) is a skeletal muscular disorder that is mainly characterized by progressive weakness of facial, scapular, and humeral muscles. Symptoms usually start within the second and third decade of life and with disease progression other muscles can become affected as well [1, 2]. Skeletal muscle pathology in patients with FSHD is caused by failure of epigenetic repression of the retrogene *DUX4*, encoding a transcription factor involved in zygotic genome activation that is silenced in most somatic tissues [3, 4]. The transcriptional changes induced by mis-expression of DUX4 in skeletal muscles ultimately lead to death of the skeletal muscle cells [5–7]. The *DUX4* gene is encoded within the D4Z4 repeat array, a macrosatellite repeat array located on chromosome 4q35 that consists of 8–100 repeat units in non-affected individuals. A prerequisite for the expression of DUX4 is the presence of a polyadenylation signal to stabilize the mRNA transcript in somatic cells that is only present on 4qA alleles [8]. Ninety-five percent of FSHD patients (FSHD1) carry a contracted D4Z4 repeat array of 1–10 units on a permissive 4qA allele [8]. This leads to epigenetic derepression of DUX4, facilitated by reduced DNA methylation levels, loss of repressive chromatin modifications, and gain of activating chromatin modifications at the D4Z4 repeat array [9–12]. In 5% of cases, FSHD is caused by digenic inheritance of a permissive 4qA allele (8-20 D4Z4 units) and a genetic variant in an epigenetic repressor of the D4Z4 repeat array (FSHD2), mostly in structural maintenance of chromosomes flexible hinge domain-containing 1 (SMCHD1) [13], a chromatin modifier involved in different processes including the maintenance of DNA methylation and X chromosome inactivation [14, 15]. The loss of SMCHD1 at the D4Z4 repeat array also leads to derepression of DUX4. Consequently, FSHD1 and FSHD2 patients show the same clinical features [16].

To study epigenetic regulation of the D4Z4 repeat array in vivo, transgenic mice carrying a FSHD-sized D4Z4 repeat array (D4Z4-2.5 mice; these mice carry a repeat array of 2.5 units) and a control-sized D4Z4 repeat array (D4Z4-12.5 mice; these mice have a repeat array of 12.5 units) were generated [17]. In hemizygous D4Z4-12.5 mice, DUX4 RNA expression is limited to the germline; in somatic tissues DUX4 is epigenetically repressed. In hemizygous D4Z4-2.5 mice, DUX4 RNA is expressed in skeletal muscles and other somatic tissues. In addition, D4Z4-2.5 mice present with loss of DNA methylation and H3K9me3 at the D4Z4 repeat array compared to D4Z4-12.5 mice, showing that transgenic FSHD mice have some mechanistic similarities regarding regulating DUX4 repression as described in humans. However, DUX4 expression in skeletal muscles of D4Z4-2.5 mice is low, consequently these mice do not develop a muscle phenotype [17]. To study the role of epigenetic modifiers in FSHD, we previously crossed D4Z4-2.5 mice with mice haploinsufficient for *Smchd1* (Smchd1^MommeD1^ mice) [18], a genetic constitution mimicking the presence of FSHD1 and FSHD2 in some affected individuals [19]. D4Z4-2.5/Smchd1^MommeD1^ mice present with a skin phenotype, reduced body weight and a short life expectancy. *Smchd1* haploinsufficiency further increased DUX4 expression levels in myoblast and myotube cultures derived from the extensor digitorum longus (EDL) muscle and in different somatic tissues including the muzzle skin and the thymus. In addition, haploinsufficiency for *Smchd1* caused a reduction in DNA methylation levels at the D4Z4 repeat array and a lower chromatin compaction score which represents the level of H3K9me3, a repressive histone mark, divided by the level of H3K4me2, an active histone mark. The H3K9me3:H3K4me2 ratio is generally lower in FSHD patients in comparison to control individuals [20]. In contrast, DUX4 transcript levels in skeletal muscles were not affected by *Smchd1* haploinsufficiency and D4Z4-2.5/Smchd1^MommeD1^ mice did not show a skeletal muscle phenotype [18].

Recently, DNA methyltransferase 3B (DNMT3B) was identified as a disease gene and disease modifier in two FSHD families [21]. DNMT3B is a de novo DNA methyltransferase crucial for the establishment of DNA methylation during early development [22]. The study of van den Boogaard et al. [21] showed that in one family carriers of the heterozygous DNMT3B missense variant (c.1579T>C) had reduced DNA methylation levels at the D4Z4 repeat array and were more likely to develop FSHD in comparison to other family members carrying an identical permissive 4qA allele of 9 D4Z4 units. In the other family, digenic inheritance of a heterozygous DNMT3B missense variant (c.2072C>T) and a permissive 4qA allele of 13 D4Z4 units induced hypomethylation at the D4Z4 repeat array but only one of the two family members was diagnosed with FSHD. DNA methyltransferases including DNMT3B are enriched at the D4Z4 repeat array [23]; however, the exact role of DNMT3B on DUX4 repression has not been studied. In this study, we examined the modifying role of Dnmt3b on epigenetic repression of DUX4 in transgenic D4Z4 mice and in mouse embryonic stem cells (mESCs) derived from D4Z4-2.5 mice. To study DUX4 regulation in vivo, hemizygous D4Z4-2.5 mice were cross-bred with mice carrying a heterozygous splice site mutation at the 5’ splice site of intron 13 in *Dnmt3b* leading to in-frame exon 13 skipping (Dnmt3b^MommeD14^ mice) [24]. This way, we mimicked the presence of FSHD1 and FSHD2 in one of the affected families with 9 D4Z4 units and a DNMT3B missense variant disrupting the C2C2-type zinc-finger motif in the ATRX-Dnmt3-Dnmt3L (ADD) domain. We chose the D4Z4-2.5 mouse model for these experiments and not the D4Z4-12.5 mouse model, as previous data from our laboratory showed that DUX4 expression was not enhanced or activated in D4Z4-12.5/Smchd1^MommeD1^ mice (unpublished data). We hypothesized that the D4Z4 repeat in FSHD mice can only be affected by these epigenetic factors if the D4Z4 repeat is already more susceptible to D4Z4 derepression, like the repeat in D4Z4-2.5 mice that present with DUX4 expression in all tissues. In contrast, mice carrying the D4Z4-12.5 repeat only show DUX4 expression in the testis [17]. In-frame skipping of exon 13 in Dnmt3b^MommeD14^ mice results in the loss of 15 amino acids close to the ADD domain which seems to make the protein more susceptible to degradation and the formation of aggregates as shown by size-exclusion chromatography experiments using recombinant proteins [24]. Homozygous Dnmt3b^MommeD14^ mice show a reduction in body weight, a distorted sex distribution, and reduced DNA methylation levels at the X-linked *Hprt* gene and telocentric repeats. This mouse model was obtained from a mutagenesis screen that aimed to identify novel epigenetic regulators of metastable epialleles and is a mouse model for Immunodeficiency, Centromeric instability, and Facial anomalies (ICF) syndrome when used in the homozygous state as bi-allelic mutations in DNMT3B cause ICF type 1 syndrome [24–27].

In this study, we present a detailed epigenetic, transcriptional, pathological, and immunological characterization of the role of Dnmt3b in D4Z4-2.5 mice. While Dnmt3b binds to and regulates the DUX4 transgene in D4Z4-2.5 mESCs, in skeletal muscles little effect of the Dnmt3b^MommeD14^ variant was observed. In contrast, secondary lymphoid organs are more sensitive to the variants in *Smchd1* and *Dnmt3b* providing a possible molecular basis for a direct involvement of the immune system in FSHD pathology.

## Methods

### Breeding and genotyping of mice

Mice were kept at the animal facility of the Leiden University Medical Center (LUMC). All experiments were carried out according to Dutch law and Leiden University guidelines and were approved by the National and Local Animal Experiments Committees. The D4Z4-2.5, Dnmt3b^MommeD14^ and Smchd1^MommeD1^ mouse models were generated before [17, 24, 25]. D4Z4-2.5/Dnmt3b^MommeD14^ mice were obtained by cross-breeding hemizygous female D4Z4-2.5 mice on a C57BL6/J background with heterozygous male Dnmt3b^MommeD14^ mice on a FVB/NJ background. D4Z4–2.5/Smchd1^MommeD1^ mice were obtained by cross-breeding female hemizygous D4Z4-2.5 mice with male heterozygous Smchd1^MommeD1^ mice, both on a C57BL6/J background. Mice were housed in individually ventilated cages with a standard 12 h/12 h light/dark cycle. Standard rodent chow and water were available ad libitum**.** The sex of the mice was recorded at 15 days by visual inspection and confirmed during dissection or during weaning. Offspring was genotyped by PCR analysis on ear DNA. To detect the D4Z4-2.5 transgene, the following primers were used: 5′-TGGTCCGTGAAGACATGTGT-3′ and 5′-GAGCCCTCAGAGAAGTGGG-3′. For the Dnmt3b^MommeD14^ mutation, the following primers were used: 5′-ATGAGGAGAGCCGAGGTGAT-3′ and 5′-ACAGCTGACACTGCCCTCTT-3′. For the Dnmt3b^MommeD14^ mutation, PCR products were digested with a *Rsa*I restriction enzyme (New England Biolabs; Bioké, Leiden, the Netherlands). To detect the Smchd1^MommeD1^ mutation, the following primers were used: 5′-CAATAGGTCCCCCTCATCAA-3′ and 5′-CAGTGGCCAACCACATAACA-3′. An internal probe with the sequence 5′-CAACATAACAGCACAACCAAAGCTG-3′ was added to the PCR mix and afterwards PCR products were melted using the Lightscanner (Idaho Technology Inc., Salt Lake City, USA).

### Culturing of mESCs and siRNA transfection

mESCs from D4Z4-2.5 mice were generated from E3.5 blastocysts after natural mating. mESCs (D4Z4-2.5 B4 and B6 cell lines) were maintained on a 0.1% gelatin coating and mitotically inactivated mouse embryonic fibroblasts (MEFs) in mESC medium (Knockout DMEM Gibco; Thermo Fisher Scientific, Bleiswijk, the Netherlands), 10% fetal bovine serum (FBS; Biowest, VWR international B.V., Amsterdam, the Netherlands), 2 mM L-glutamine (Gibco; Thermo Fisher Scientific, Bleiswijk, the Netherlands), 1 mM sodium pyruvate (Gibco; Thermo Fisher Scientific, Bleiswijk, the Netherlands), 1% non-essential amino acids solution (Gibco; Thermo Fisher Scientific, Bleiswijk, the Netherlands), 0.1 mM 2-mercaptoethanol (Gibco; Thermo Fisher Scientific, Bleiswijk, the Netherlands), and 1000 U/ml leukemia inhibitory factor (Merck, Amsterdam, the Netherlands). For siRNA transfections, mESCs were cultured in a 12 well culture dish on 0.1% gelatin without MEFs. Pre-designed siRNA SMARTpool for Dnmt3b (M-044164-01-0005, Dharmacon; Horizon Discovery Ltd., Cambridge, UK) and luciferase siRNA 1 (D-002050-01-20, Dharmacon; Horizon Discovery Ltd., Cambridge, UK) at a concentration of 40 nM and 2 μl RNAiMAX transfection reagent (Thermo Fisher Scientific, Bleiswijk, the Netherlands) were used. Transfection medium was changed to mESC medium the next day and cells were harvested after 48 h.

### Isolation and culturing of myoblasts derived from the EDL muscle

The EDL muscle was removed from mice and incubated at 37 °C for 105 min in 0.2% collagenase (Sigma Aldrich, Zwijndrecht, the Netherlands) in DMEM medium (Gibco; Thermo Fisher Scientific, Bleiswijk, the Netherlands) containing 1% penicillin-streptomycin (P/S) (Sigma Aldrich, Zwijndrecht, the Netherlands). Muscle fibers were further dissociated with a glass Pasteur pipette with a smooth end and were transferred to a 6 well culture dish coated with Matrigel (BD Biosciences, Vianen, the Netherlands) in DMEM medium supplemented with 30% FBS, 10% horse serum, 1% P/S, 1% chicken embryo extract, and 2.5 ng/ml fibroblast growth factor (all from Thermo Fisher Scientific, Bleiswijk, the Netherlands). Fibers were removed and attached myoblasts were trypsinized and plated in new Matrigel-coated plates. Myoblasts were harvested for RNA or differentiated by changing medium to DMEM with 2% horse serum and 1% P/S. Differentiated myotubes were harvested 72 h after the start of differentiation.

### RNA isolation, cDNA synthesis, and Dnmt3b RT-PCR

Total RNA was isolated with the miRNeasy kit (Qiagen, Venlo, the Netherlands) according to the manufacturer’s instructions. A DNase treatment on the column for 30 min at RT was included. RNA concentrations were measured with the Nanodrop ND-1000 spectrophotometer (Thermo Fisher Scientific, Bleiswijk, the Netherlands). 1–3 μg RNA was reverse transcribed according to the manufacturer’s instructions using the RevertAid H Minus First Strand cDNA synthesis kit with Oligo(dT) primers (Thermo Fisher Scientific, Bleiswijk, the Netherlands). The cDNA was treated with RNaseH (Thermo Fisher Scientific, Bleiswijk, the Netherlands) for 20 min at 37 °C. The Dnmt3b^MommeD14^ variant was confirmed in D4Z4-2.5/Dnmt3b^MommeD14^ mice with a PCR spanning exons 12 and 14 with the following primers: 5′-CGAAGACGCACAACCAATGA-3′ and 5′-TTTCCACAGGACAAACAGCG-3′.

### RT-qPCR

For RT-qPCR with the CFX96 system (Bio-Rad, Veenendaal, the Netherlands), iQ SYBR Green Supermix (Biorad), 0.5 pM forward and reverse primer (Table 1) and 1:5 or 1:50 diluted cDNA were used with the following cycling conditions: 95 °C for 3 min, 40 cycles of 10 s at 95 °C and a melting temperature of 60 °C for 30 s, followed by a melting curve analysis from 65 °C to 95 °C (temperature increments of 0.5 °C). Data was analyzed with Bio-Rad CFX Manager version 3.1 (Bio-Rad, Veenendaal, the Netherlands). Obtained values were normalized to the housekeeping genes Gapdh and Rpl13a.

**Table 1:** *List of RT-qPCR primers*

Gene	Forward primer	Reverse primer
CD19	GGCTTCTACCTGTGCCAGAA	CTGACGTCTGAAGCATTCCA
Cxcl12	ACCAGTCAGCCTGAGCTACC	TAATTTCGGGTCAATGCACA
Dnmt3b	CGCAGGAAAGATTGGAACAT	CGTTGCAATTCCATCAAACA
DUX4	CCCAGGTACCAGCAGACC	TCCAGGAGATGTAACTCTAATCCA
Gapdh	TCCATGACAACTTTGGCATTG	TCACGCCACAGCTTTCCA
IFNγ	CTTCTTCAGCAACAGCAAGG	TGAGCTCATTGAATGCTTGG
IL-1β	CTTTCCCGTGGACCTTCC	CATCATCATCCCATGAGTCAC
IL-6	TGTGCAATGGCAATTCTGAT	CTCTGAAGGACTCTGGCTTTG
Mef2c	TCCATCAGCCATTTCAACAA	GTTACAGAGCCGAGGTGGAG
Podoplanin	ATTGTGACCCCAGGTACAGG	GCTGAGGTGGACAGTTCCTC
Rpl13a	TGCTGCTCTCAAGGTTGTTC	TTCTCCTCCAGAGTGGCTGT
TNFα	CGAGTGACAAGCCTGTAGCC	CTTTGAGATCCATGCCGTTG
Wfdc3	CTTCCATGTCAGGAGCTGTG	ACCAGGATTCTGGGACATTG

### DNA methylation analysis of the D4Z4 repeat array

Genomic DNA from the tail and spleen was bisulfite converted with the EZ DNA Methylation-Lightning kit (Zymo Research; BaseClear Lab Products, Leiden, the Netherlands) according to the manufacturer’s instructions. A PCR reaction of the pLAM region just distal to the D4Z4 repeat array was performed [28]. PCR products were next ligated into a TOPO TA vector (Thermo Fisher Scientific, Bleiswijk, the Netherlands) and the resulting ligation products were transformed into competent D5Hα bacteria by heat shock. Plasmid DNA from at least 10 colonies of transformed D5Hα bacteria was isolated and sent for Sanger sequencing.

### Dnmt3b and histone chromatin immunoprecipitation (ChIP)

mESCs were maintained on a 0.1% gelatin coating and mitotically inactivated MEFs in mESC medium. Spleen tissue was minced into small pieces. Both mESCs and splenic tissue were crosslinked with 1% formaldehyde. 125 mM glycine was added after 10 min to stop the crosslinking. Cells and tissues were lysed with cytoplasmic lysis buffer (10 mM Tris pH 8, 10 mM NaCl, 0.2% Igepal for spleen tissues and 0.5% Igepal for mESCs). The nuclei were lysed with nuclear lysis buffer (1% SDS, 10 mM EDTA, 50 mM Tris pH 8.1). Chromatin of mESCs was sheared with 15 cycles of 30 s and spleen tissue using 10 cycles of 30 s with the Bioruptor (Pico; Diagenode, Ougrée, Belgium). The fragmentation was verified on a 1% agarose gel after phenol-chloroform extraction. For the Dnmt3b ChIP, 50 μg chromatin was used as input and for each histone ChIP 3 μg chromatin was used. Per antibody, chromatin was pre-cleared with 20 μl protein A Sepharose beads (GE Healthcare, Eindhoven, The Netherlands). Pre-cleared chromatin was incubated overnight with 5 μg of one of the following antibodies: Dnmt3b (ab2851; Abcam, Cambridge, UK), IgG (PP64; Merck, Amsterdam, the Netherlands), H3K9me3 (39161; Active Motif, Carlsbad, USA), or H3K4me2 (39141; Active Motif, Carlsbad, USA). The immunoprecipitation was performed by incubating 20 μl protein A Sepharose beads with the antibody-chromatin mix for 2 h at 4 °C. Washing of the beads was performed according to a previously published protocol [29]. DNA was isolated from the beads with Chelex resin (Bio-Rad, Veenendaal, the Netherlands). qPCR to amplify the D4Z4 repeat was performed with the following primers: 5′-CCGCGTCCGTCCGTGAAA-3′ and 5′-TCCGTCGCCGTCCTCGTC-3′ [9].

### Western blot analysis

Testis and mESCs were homogenized in lysis buffer (8 M Urea, 10% glycerol, 1% SDS, 5 mM DTT, 10 mM Tris pH 7.4). The lysates were sonicated for 10 cycles of 30 s with the Bioruptor (Pico; Diagenode, Ougrée, Belgium). After centrifugation, the supernatant was kept and protein concentrations were determined using the Pierce™ BCA Protein Assay Kit (Thermo Fisher Scientific, Bleiswijk, the Netherlands). Equal amounts of proteins were separated on a NuPAGE™ 4–12% Bis-Tris Protein Gel (Thermo Fisher Scientific, Bleiswijk, the Netherlands) and transferred to a Immobilon-FL PVDF membrane (Merck, Amsterdam, the Netherlands). The membrane was incubated in 4% skim milk/PBS overnight at 4 °C with the following antibodies: rabbit-anti-Dnmt3b antibody (ab2851; Abcam, Cambridge, UK; 1:600 dilution) and mouse-anti-emerin (NCL-emerin, clone 4G5; Novocastra; Leica Biosystems B.V., Amsterdam, the Netherlands, 1:375 dilution). The following day, the membrane was incubated with the secondary antibodies donkey-anti-rabbit-IRdye 800cw (1:5000 dilution) and donkey-anti-mouse-IRdye 680RD (1:5000 dilution) (Li-Cor, Bad Homburg, Germany) for 1 h at RT in 4% skim milk/PBS. The membrane was scanned with the Odyssey CLx imager (Li-Cor, Bad Homburg, Germany).

### Flow cytometry analysis

The spleen was dissected from euthanized mice. Immune cells were separated from stromal tissues by mincing the organs and straining the tissue through a 70 μm cell strainer. Single cells were stained with cell surface antibodies (Table 2) for 20 min at 4 °C in FACS buffer (DPBS with 1% FBS and 0.05% sodium azide). Cells were fixed for 45 min at RT with True-Nuclear Fix solution (Biolegend, London, UK). For intracellular staining with FoxP3 (Table 2) for 30 min at RT, the True-Nuclear Permeabilization Buffer (Biolegend, London, UK) was used. Cells were measured with the Canto I (Becton Dickinson B.V., Vianen, the Netherlands) and analyzed with FlowJo software v10.6.1 (Becton Dickinson B.V., Vianen, the Netherlands).

**Table 2:** List of surface and intracellular (FoxP3) anti-mouse antibodies that were used for flow cytometry

Product	Clone	Company
AF647 anti-mouse FoxP3	MF-14	Biolegend
APC anti-mouse CD44	IM7	Biolegend
Pacific Blue anti-mouse CD8a	53-6.7	Biolegend
Pacific Blue anti-mouse CD11b	M1/70	Biolegend
PE anti-mouse CD11c	N418	Biolegend
PE anti-mouse CD19	6D5	Biolegend
PE anti-mouse CD25	PC61	Biolegend
PE/Cy7 anti-mouse CD90.2	53-2.1	Biolegend
PE/Cy7 anti-mouse Ly-6C	HK1.4	Biolegend
PerCP anti-mouse CD4	GK1.5	Biolegend
PerCP anti-mouse Ly6G	1A8	Biolegend
FITC anti-mouse F4/80	BM8	eBioscience

### H&E staining

Skeletal muscles were dissected from euthanized mice, embedded in O.C.T. Compound (Tissue-Tek) and frozen in cooled isopentane. Cryosections of 7 μm were made and stained with hematoxylin, followed by eosin staining. Cryosections were dehydrated using increasing ethanol concentrations and xylene. Stained skeletal muscle sections were analyzed by light microscopy (Leica Microsystems B.V., Amsterdam, the Netherlands).

### Statistical analysis

Statistical tests were performed with GraphPad Prism software (version 8; GraphPad Software, Inc., La Jolla, USA). For all figures, the statistical tests that were performed are described in the figure legends. Values of *P* < 0.05 were considered significant.

## Results

### Dnmt3b affects DUX4 expression in mESCs, but the Dnmt3b^MommeD14^ variant does not induce a more severe phenotype in FSHD mice

**Fig. 1:**
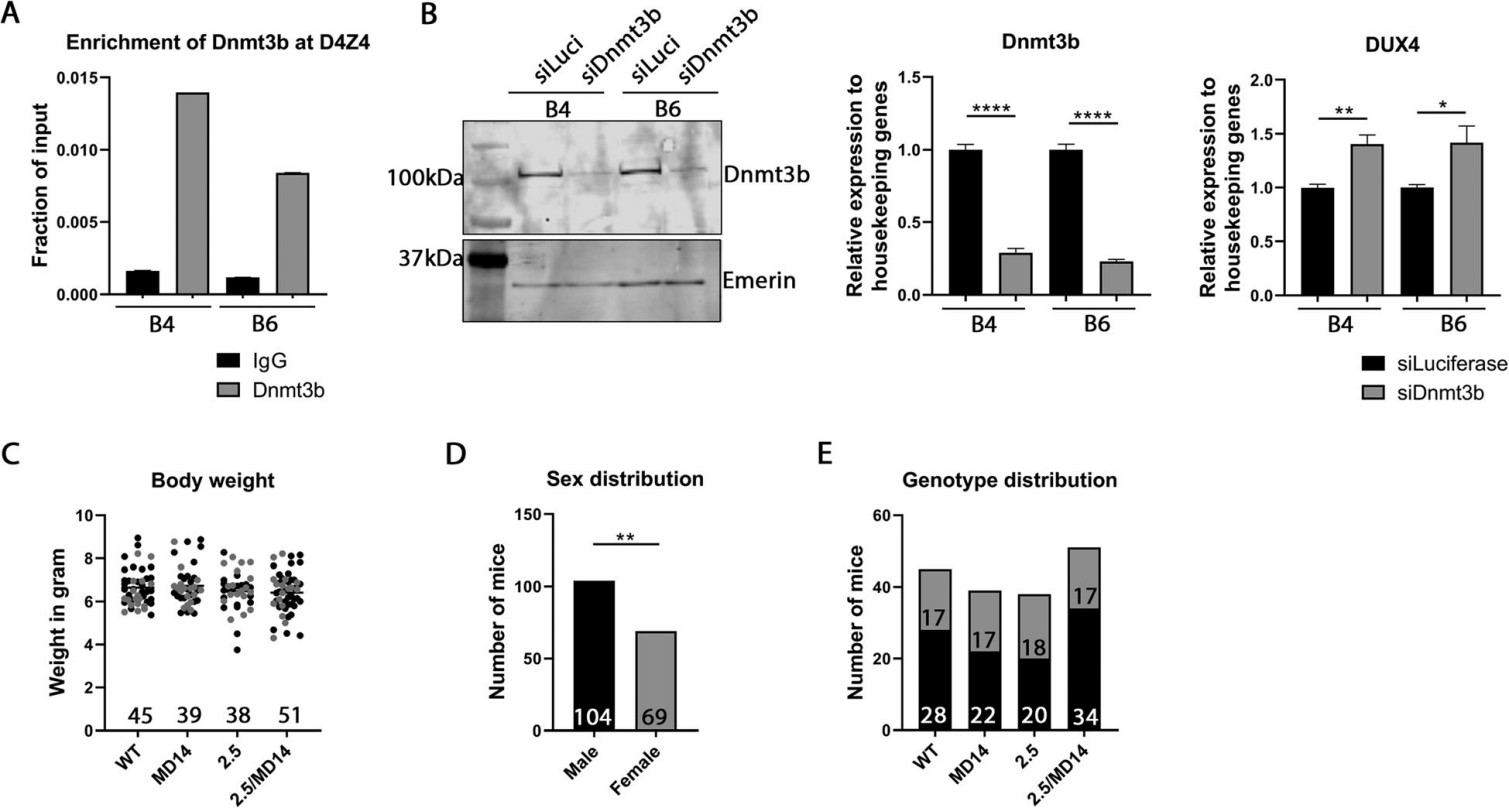
Dnmt3b affects DUX4 expression in mESCs, but the Dnmt3b^MommeD14^ variant does not induce a more severe phenotype in FSHD mice. **a** Dnmt3b ChIP-qPCR analysis showed that Dnmt3b is enriched at the D4Z4 repeat array in mESCs. The error bars represent the standard error of the mean (SEM) for two technical replicates. **b** A successful knockdown of *Dnmt3b* was confirmed by western blot and RT-qPCR in the B4 and B6 cell lines. DUX4 transcript levels were significantly enhanced as determined by a Student’s *t* test. *P < 0.05; **P < 0.01; ****P < 0. 0001. Error bars represent the SEM from five technical replicates. **c** Body weight at postnatal day 15 was not changed between all genotypes. Each dot represents a single mouse (male mice in black; female mice in grey) and the *N* is depicted per genotype. Statistical analysis was performed by one-way ANOVA. WT = wild-type; 2.5 = D4Z4-2.5; MD14 = Dnmt3b^MommeD14^. **d** Sex distribution was significantly disturbed; fewer female mice were born from the cross-breedings. Statistical analysis was performed using a Pearson’s chi-squared test. **P < 0.01. **e** Genotype distribution (male mice in black; female mice in grey) was not disturbed as tested by a Pearson’s chi-squared test. The *N* for male and female mice is depicted for each genotype. WT = wild-type; 2.5 = D4Z4-2.5; MD14 = Dnmt3b^MommeD14^

The D4Z4-2.5 mouse model that we previously generated carries a transgenic FSHD1-sized D4Z4 repeat array [17]. To verify that murine Dnmt3b can influence the transgene, we first performed experiments in mESCs derived from transgenic D4Z4-2.5 mice as Dnmt3b is mostly involved in the establishment of DNA methylation during early development. A Dnmt3b ChIP-qPCR was performed in two different D4Z4-2.5 mESC lines (B4 and B6) to determine whether murine Dnmt3b binds to the D4Z4 repeat array. In both cell lines, we confirmed that murine Dnmt3b is enriched at the D4Z4 repeat array (Fig. 1a). Next, siRNA knockdowns of *Dnmt3b* were performed in the same cell lines. A successful knockdown was confirmed by measuring *Dnmt3b* transcript and protein levels. DUX4 transcript levels were enhanced after *Dnmt3b* knockdown in both cell lines (Fig. 1b). We concluded that Dnmt3b can influence DUX4 expression and continued with in vivo studies in transgenic mice by cross-breeding hemizygous female D4Z4-2.5 mice with heterozygous male Dnmt3b^MommeD14^ mice [24]. Approximately 170 pups were born from these cross-breedings. Of all pups, sex, genotype, and body weight at postnatal day 15 were recorded. No differences in body weight were found when comparing wild-type, Dnmt3b^MommeD14^, D4Z4-2.5, and D4Z4-2.5/Dnmt3b^MommeD14^ mice (Fig. 1c). However, fewer female mice were born from these cross-breedings (*P* = 0.0078) (Fig. 1d). The genotype distribution was not disturbed as we observed a Mendelian inheritance pattern (Fig. 1e). Finally, D4Z4-2.5/Dnmt3b^MommeD14^ mice did not show a runting phenotype or a short life expectancy which we reported before in D4Z4-2.5/Smchd1^MommeD1^ mice. D4Z4-2.5/Dnmt3b^MommeD14^ mice generally appeared to be healthy, except for a delayed opening of their eyes at postnatal day 15, which we also observed for the D4Z4-2.5 mice born from these cross-breedings. This is likely due to a skin phenotype consisting of hyperkeratosis, as previously found in D4Z4-2.5/Smchd1^MommeD1^ mice and their D4Z4-2.5 littermates [18].

### The Dnmt3b^MommeD14^ variant affects Dnmt3b protein levels but not Dnmt3b mRNA levels

**Fig. 2:**
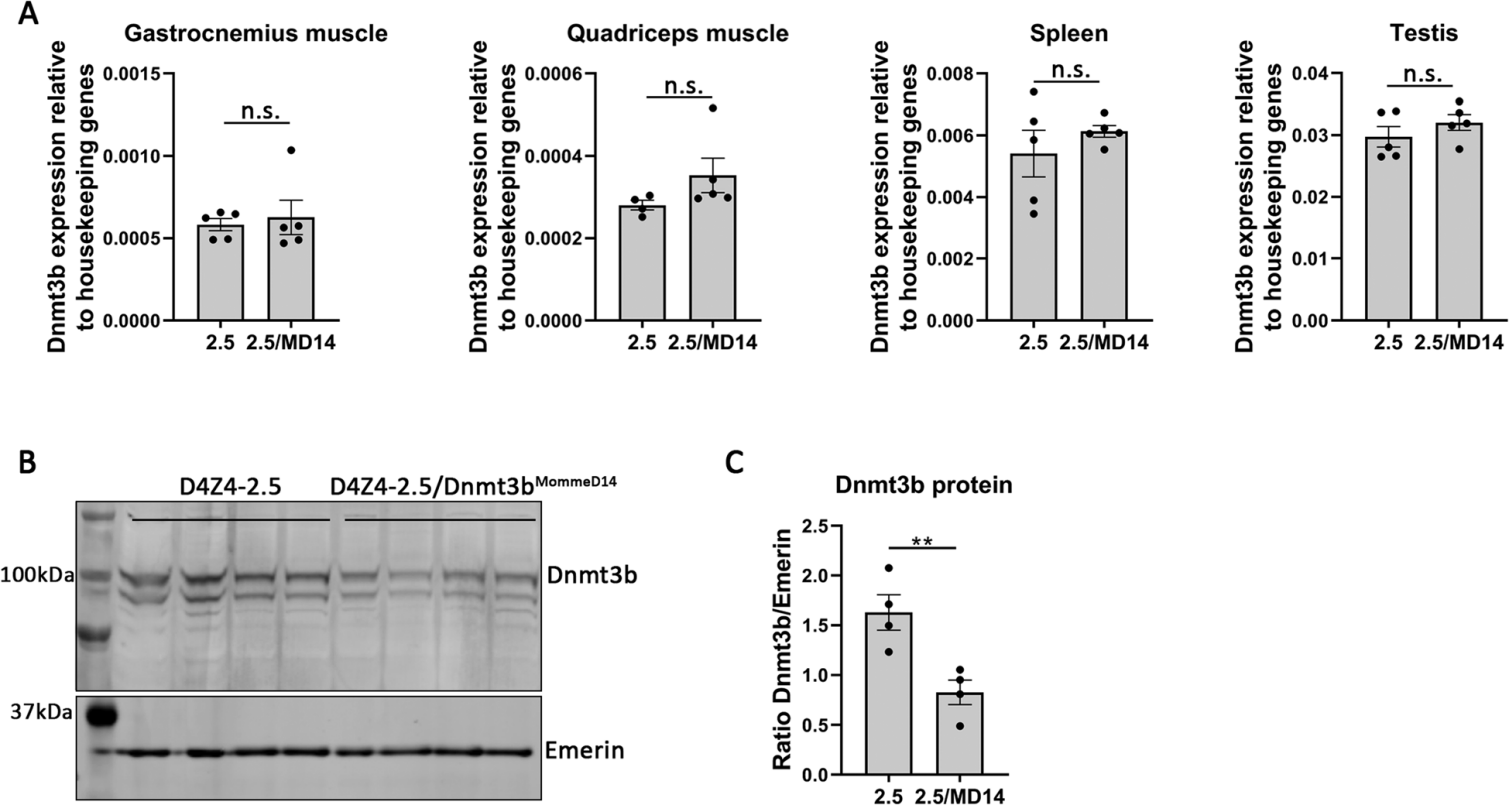
The Dnmt3b^MommeD14^ variant affects Dnmt3b protein levels but not Dnmt3b mRNA levels. *Dnmt3b* transcript levels were not affected by the Dnmt3b^MommeD14^ variant as measured by RT-qPCR in different skeletal muscles and non-muscle tissues (postnatal day 15). Each dot represents one mouse, and the error bars denote the SEM from the biological replicates. Statistical analysis was performed using a Student’s *t* test (testis, spleen) or Mann-Whitney *U* test (quadriceps and gastrocnemius muscle). n.s. = not significant. 2.5 = D4Z4-2.5; MD14 = Dnmt3b^MommeD14^. **b** Dnmt3b protein levels were slightly reduced in the testis of D4Z4-2.5/Dnmt3b^MommeD14^ mice at postnatal day 15. Emerin was used as a loading control. **c** Quantification of Dnmt3b protein levels of the upper and lower band showed a significant reduction in the Dnmt3b/Emerin ratio in the testis of D4Z4-2.5/Dnmt3b^MommeD14^ mice as determined by a Student’s *t* test. Each dot represents one mouse, and the error bars denote the SEM from the biological replicates. **P < 0.01. 2.5 = D4Z4-2.5; MD14 = Dnmt3b^MommeD14^

The Dnmt3b^MommeD14^ mutation is a hypomorphic variant that leads to in-frame skipping of exon 13 [24]. As expected, the in-frame exon skipping mutation did not affect *Dnmt3b* transcript levels in skeletal muscles and non-muscle tissues (Fig. 2a). A western blot for Dnmt3b was performed on the testis as this tissue still contains high protein levels of Dnmt3b after early development. The Dnmt3b protein levels were reduced in D4Z4-2.5/Dnmt3b^MommeD14^ mice in comparison to D4Z4-2.5 mice at postnatal day 15 (Fig. 2b). Quantification of Dnmt3b by correcting for Emerin showed that the Dnmt3b^MommeD14^ variants leads to a significant reduction in Dnmt3b protein levels (*P* = 0.0099, Fig. 2c).

### The Dnmt3b^MommeD14^ variant does not induce DUX4 expression or skeletal muscle pathology in D4Z4-2.5 mice

**Fig. 3:**
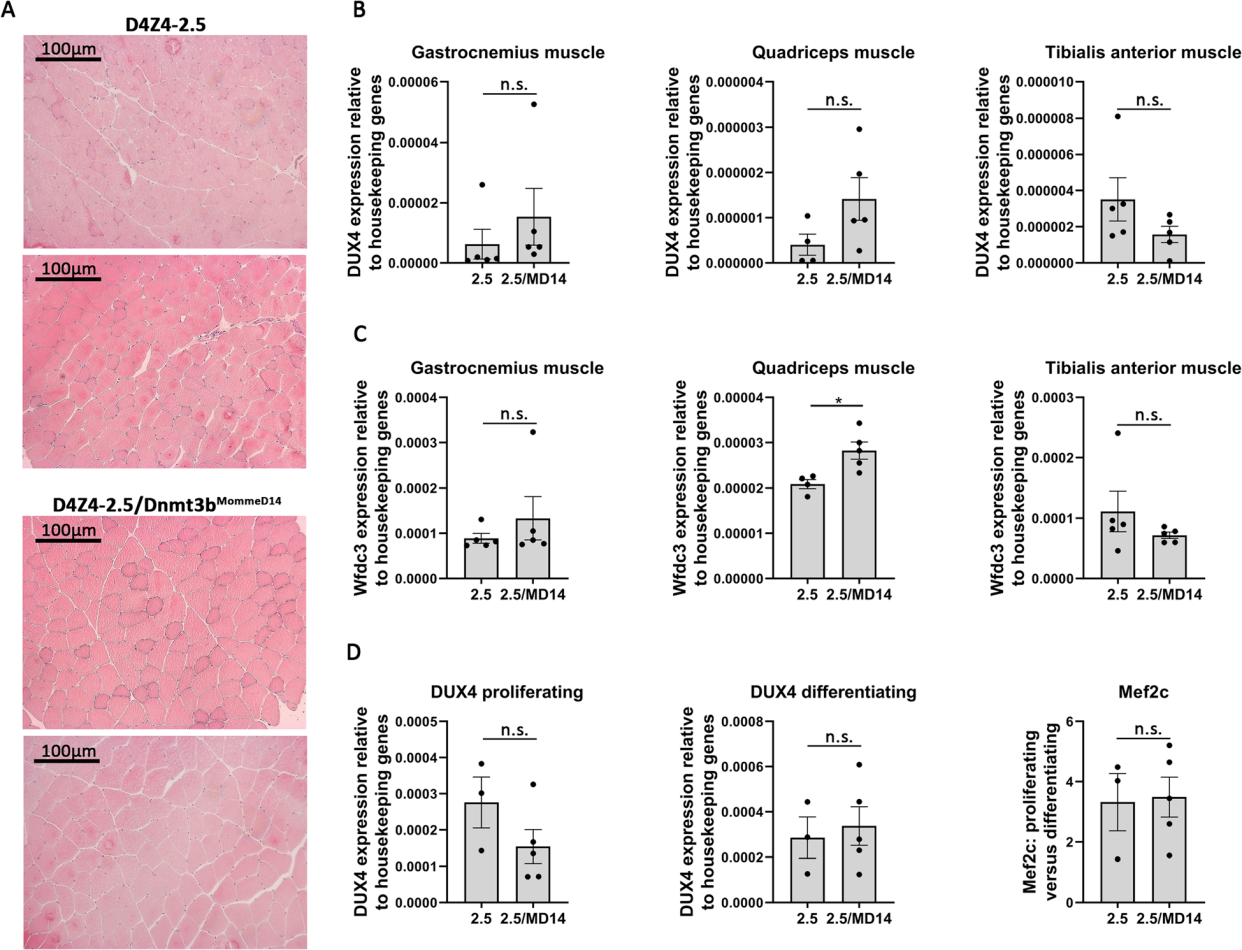
The Dnmt3b^MommeD14^ variant does not induce DUX4 expression or skeletal muscle pathology in D4Z4-2.5 mice. **a** Representative H&E-stained cross-sections of the quadriceps muscle (× 100 magnification) showed no skeletal muscle pathology in D4Z4-2.5 and D4Z4-2.5/Dnmt3b^MommeD14^ mice at 24 weeks of age. **b** DUX4 transcript levels in different skeletal muscles were low and not affected by the Dnmt3b^MommeD14^ variant at postnatal day 15. Statistical analysis was performed using a Student’s *t* test (quadriceps and tibialis anterior muscle) or a Mann-Whitney *U* test (gastrocnemius muscle). Each dot represents a single mouse, and the error bars denote the SEM from the biological replicates. n.s. = not significant. 2.5 = D4Z4-2.5; MD14 = Dnmt3b^MommeD14^. **c** Target gene expression of *Wfdc3* showed a slight upregulation in the quadriceps muscle of D4Z4-2.5/Dnmt3b^MommeD14^ mice at postnatal day 15. Statistical analysis was performed using a Student’s *t* test (quadriceps and tibialis anterior muscle) or a Mann-Whitney *U* test (gastrocnemius muscle). Each dot represents a single mouse, and the error bar signifies the SEM from the biological replicates. *P < 0.05. n.s. = not significant. 2.5 = D4Z4-2.5; MD14 = Dnmt3b^MommeD14^. **d** DUX4 and *Mef2c* expression as measured by RT-qPCR in proliferating and differentiating muscle cells derived from the EDL muscle (postnatal day 15). The Dnmt3b^MommeD14^ variant does not affect the DUX4 expression in the EDL-derived muscle cells. *Mef2c* transcript levels were measured as a marker for differentiation toward myotubes. Per mouse, the ratio between proliferating versus differentiating cells is depicted. Each dot represents a single mouse, and the error bars denote the SEM from the biological replicates. Differences in DUX4 expression were tested using a Student’s *t* test. n.s. = not significant. 2.5 = D4Z4-2.5; MD14 = Dnmt3b^MommeD14^

In skeletal muscles of D4Z4-2.5 mice DUX4 transcript levels are low; consequently, these mice do not develop skeletal muscle pathology [17]. Haploinsufficiency for *Smchd1* also did not lead to histological abnormalities at postnatal day 15, even though DUX4 transcript levels were enhanced in the quadriceps muscle [18]. Because of severe body weight loss, we could not analyze the skeletal muscles during adulthood. The D4Z4-2.5/Dnmt3b^MommeD14^ mice do not show a severe phenotype, therefore we could assess whether skeletal muscle pathology developed during adulthood. However, in both D4Z4-2.5 and D4Z4-2.5/Dnmt3b^MommeD14^ mice, we found no signs of muscle pathology in the triceps muscle (data not shown) and the quadriceps muscle at the age of 24 weeks as visualized by hematoxylin and eosin (H&E) staining (Fig. 3a). In addition, no enhanced DUX4 transcript levels were measured in different skeletal muscles of D4Z4-2.5/Dnmt3b^MommeD14^ mice in comparison to D4Z4-2.5 mice (Fig. 3b). Only transcript levels of the DUX4 target gene *Wfdc3* showed a slight upregulation in the quadriceps muscle of D4Z4-2.5/Dnmt3b^MommeD14^ mice (Fig. 3c). However, in comparison to wild-type and Dnmt3b^MommeD14^ mice, *Wfdc3* expression was not upregulated in mice with a D4Z4 transgene, showing that the low DUX4 expression in the skeletal muscles does not activate this DUX4 target gene (Fig. S1A). As DUX4 expression is typically higher in myoblast cultures, we next derived muscle cells from the EDL muscle and differentiated these towards myotubes ex vivo. In both proliferating and differentiating cells, no differences in DUX4 transcript levels were measured by RT-qPCR between D4Z4-2.5 and D4Z4-2.5/Dnmt3b^MommeD14^ mice. Differentiation of myoblasts to myotubes was confirmed by *Mef2c* expression (Fig. 3d). Taken together, our results show that skeletal muscles of D4Z4-2.5 mice remain unaffected by the Dnmt3b^MommeD14^ variant.

### In non-muscular tissues, enhanced DUX4 expression in D4Z4-2.5/Dnmt3b^MommeD14^ mice is limited to the secondary lymphoid organs

**Fig. 4:**
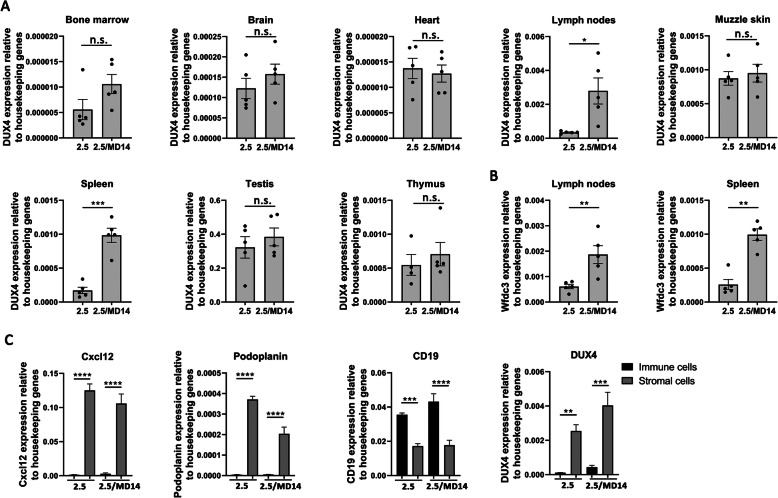
In non-muscular tissues, enhanced DUX4 expression in D4Z4-2.5/Dnmt3b^MommeD14^ mice is limited to the secondary lymphoid organs. **a** DUX4 transcript levels were enhanced as measured by RT-qPCR in the inguinal lymph nodes and the spleen of D4Z4-2.5/Dnmt3b^MommeD14^ mice (postnatal day 15) while the other tested tissues remained unaffected. Each dot represents one mouse, and the error bars denote the SEM of the biological replicates. Statistical analysis was performed with a Student’s *t* test (brain, heart, lymph nodes, muzzle skin, spleen, testis) or a Mann–Whitney *U* test (bone marrow, thymus). *P < 0.05; ***P < 0.001. n.s. = not significant. 2.5 = D4Z4-2.5; MD14 = Dnmt3b^MommeD14^. **b** Transcript levels of the target gene *Wfdc3* were upregulated in the inguinal lymph nodes and the spleen of D4Z4-2.5/Dnmt3b^MommeD14^ mice at postnatal day 15 as measured by RT-qPCR. Each dot represents one mouse, and the error bars denote the SEM of the biological replicates. Statistical analysis was performed using a Student’s *t* test (lymph nodes) or Mann–Whitney *U* test (spleen). **P < 0.01. 2.5 = D4Z4-2.5; MD14 = Dnmt3b^MommeD14^. **c** With RT-qPCR we showed that *Cxcl12* and *Podoplanin* transcripts (stromal cell markers) were highly enriched in the stromal cell fraction while *CD19* transcripts (B cell marker) were enriched in the immune cell fraction, confirming a successful separation of both fractions. DUX4 transcripts were highly enriched in the stromal cells, while the immune cells showed a low expression. In both fractions, DUX4 transcript levels were elevated by the Dnmt3b^MommeD14^ variant (not significant). Statistical analysis was performed by one-way ANOVA. Each bar represents at least four biological replicates, and the error bars denote the SEM. **P < 0.01; ***P < 0.001; ****P < 0.0001. n.s. = not significant. 2.5 = D4Z4-2.5; MD14 = Dnmt3b^MommeD14^

Previously, we showed that haploinsufficiency for *Smchd1* led to a thickening of the epidermis in all D4Z4-2.5 mice and a reduced size of the thymus in D4Z4-2.5 mice with a low body weight. In both tissues, DUX4 transcript levels were significantly enhanced at postnatal day 15 in comparison to D4Z4-2.5 mice without the Smchd1^MommeD1^ variant while other non-skeletal muscle tissues remained unaffected [18]. In the D4Z4-2.5/Dnmt3b^MommeD14^ mice, no additional phenotypes in the skin or thymus were observed compared to the D4Z4-2.5 mice. In line with this, DUX4 transcript levels at postnatal day 15 in the muzzle skin and thymus were not altered. Furthermore, the Dnmt3b^MommeD14^ variant did not affect DUX4 expression in the brain, bone marrow, heart, or testis [Fig. 4a]. Interestingly, we measured significantly higher DUX4 transcript levels in the inguinal lymph nodes (*P* = 0.01) and in the spleen (*P* = 0.0001), showing that secondary lymphoid organs are affected by the Dnmt3b^MommeD14^ variant. Transcript levels of the DUX4 target gene *Wfdc3* were also significantly upregulated in the inguinal lymph nodes (*P* = 0.0087) and in the spleen (*P* = 0.0079) (Fig. 4b) but not in other tissues (data not shown). In addition, *Wfdc3* expression levels in the secondary lymphoid organs were increased in comparison to wild-type and Dnmt3b^MommeD14^ mice (Fig. S1B). To determine which cells in the spleen are sensitive for the Dnmt3b^MommeD14^ variant, we performed RT-qPCR analysis for immune cell and stromal cell fractions derived from the spleen, which were separated from each other by mincing the spleen over a 70 μm filter. A successful separation of both fractions was confirmed by measuring different stromal and immune cell markers (Fig. 4c). Interestingly, DUX4 transcript levels were highly expressed in the stromal cells in both D4Z4-2.5 and D4Z4-2.5/Dnmt3b^MommeD14^ mice. In both the immune cell and the stromal cell fraction, DUX4 transcript levels were higher in D4Z4-2.5/Dnmt3b^MommeD14^ mice compared to D4Z4-2.5 mice, showing that not one specific cell type in the spleen is sensitive to the Dnmt3b^MommeD14^ variant.

### DNA methylation at the D4Z4 repeat array, but not the chromatin compaction score, is affected by the Dnmt3b^MommeD14^ variant

**Fig. 5:**
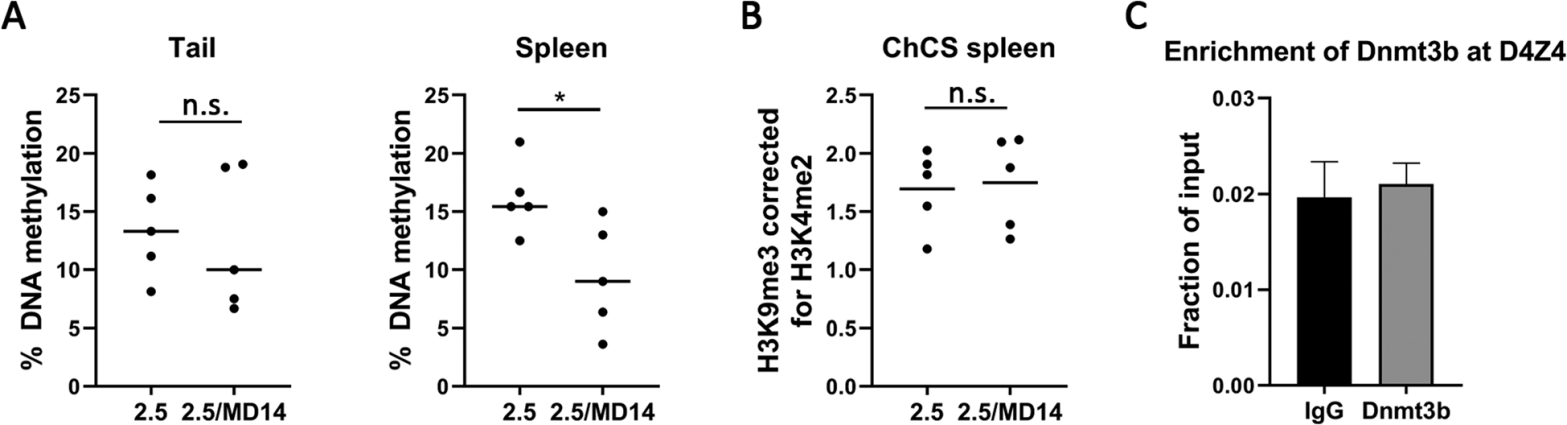
DNA methylation at the D4Z4 repeat array, but not the chromatin compaction score, is affected by the Dnmt3b^MommeD14^ variant. **a** The average DNA methylation level of 10 CpG dinucleotides just distal to the D4Z4 repeat (the FasPas region) was not changed in tail DNA of D4Z4-2.5/Dnmt3b^MommeD14^ mice (postnatal day 15). In contrast, in spleen DNA a significant reduction in DNA methylation levels was detected in D4Z4-2.5/Dnmt3b^MommeD14^ mice compared to D4Z4-2.5 mice (*P* = 0.026). Each dot represents the average DNA methylation level of 10 CpG sites per mouse. Differences between groups were compared using a Student’s *t* test. *P < 0.05. n.s. = not significant. 2.5 = D4Z4-2.5; MD14 = Dnmt3b^MommeD14^. **b** The chromatin compaction score (H3K9me3 level corrected for H3K4me2 level) was not different in the spleen of D4Z4-2.5/Dnmt3b^MommeD14^ mice compared to D4Z4-2.5 mice (postnatal day 15) as measured by ChIP-qPCR analysis. Each dot represents one mouse. Statistical analysis was performed using a Student’s *t* test. n.s. = not significant. 2.5 = D4Z4-2.5; MD14 = Dnmt3b^MommeD14^. **c** Dnmt3b is not enriched at the D4Z4 repeat in the spleen of D4Z4-2.5 mice (postnatal day 15) as determined by ChIP-qPCR. The error bars represent the SEM for two biological replicates

In D4Z4-2.5 mice, DNA methylation levels at the D4Z4 repeat array are low. These levels are further reduced when haploinsufficiency for *Smchd1* is introduced [18]. To determine the effect of the Dnmt3b^MommeD14^ variant on DNA methylation, sodium bisulfite sequencing was performed for 10 CpG dinucleotides just distal to the D4Z4 repeat array [28]. First, we measured DNA methylation levels in tail DNA, but no differences were found. As DUX4 transcript levels in the spleen were enhanced by the Dnmt3b^MommeD14^ variant, we next measured DNA methylation levels in the spleen. We found that the average DNA methylation level at the D4Z4 repeat array in DNA isolated from the spleen was significantly reduced in D4Z4-2.5/Dnmt3b^MommeD14^ mice in comparison to D4Z4-2.5 mice (*P* = 0.026) (Fig. 5a). Next, we performed H3K9me3 and H3K4me2 ChIP-qPCR analysis in the spleen to quantify the chromatin compaction score as this tissue showed changes in DNA methylation at the D4Z4 repeat array and in DUX4 expression. We previously found a lower chromatin compaction score in D4Z4-2.5/Smchd1^MommeD1^ mice in comparison to D4Z4-2.5 mice [18]. In D4Z4-2.5/Dnmt3b^MommeD14^ mice the chromatin compaction score was not altered [Fig. 5b], indicating that the Dnmt3b^MommeD14^ variant does not have a major impact on histone modifications of the D4Z4 repeat array. At last, we verified whether Dnmt3b can still bind to the D4Z4 repeat array in somatic spleen tissue. However, Dnmt3b was not enriched at the D4Z4 repeat [Fig. 5c]. Therefore, we hypothesize that the changes in DNA methylation and DUX4 expression in the spleen are caused by a failure of establishment of DNA methylation at the D4Z4 repeat during early development.

### Specific immune cell populations in the secondary lymphoid organs of D4Z4-2.5/Smchd1^MommeD1^ are disturbed

**Fig. 6:**
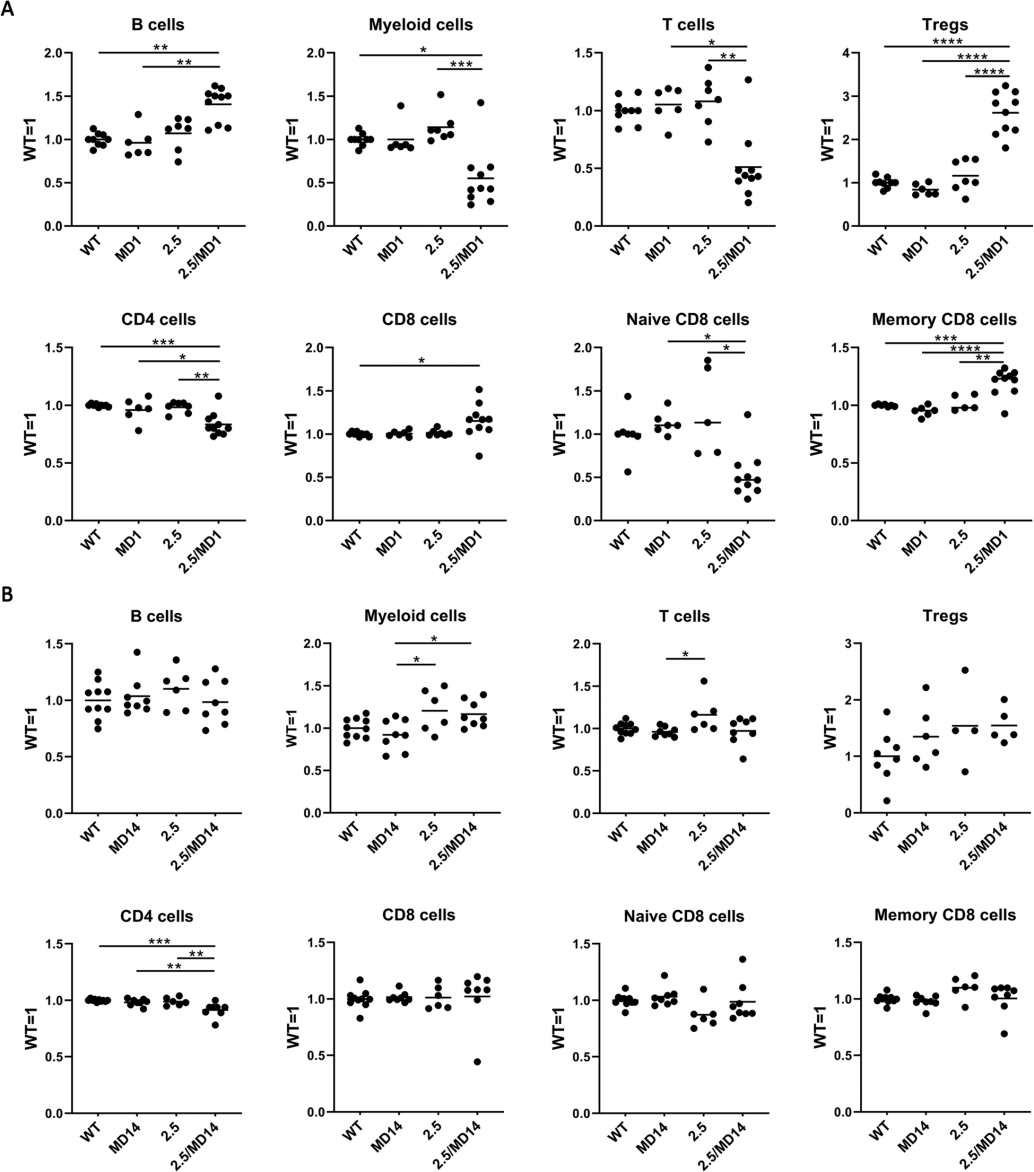
Specific immune cell populations in the secondary lymphoid organs of D4Z4-2.5/Smchd1^MommeD1^ are disturbed. Splenic immune cells from pups derived from crossbreeding hemizygous D4Z4-2.5 mice with heterozygous Smchd1^MommeD1^ mice (**a**) or from crossbreeding hemizygous D4Z4-2.5 mice with heterozygous Dnmt3b^MommeD14^ mice (**b**) were isolated, stained with different antibodies, and measured by flow cytometry. The average percentage of immune cells found in wild-type mice in each litter was set to 1 and ratios were calculated per experiment. Each dot represents one mouse (postnatal day 15). Statistical analysis was performed using one-way ANOVA. *P < 0.05; **P < 0.01; ***P < 0.001; ****P < 0.0001. Other results were not significant. 2.5 = D4Z4-2.5; MD1 = Smchd1^MommeD1^; MD14 = Dnmt3b^MommeD14^

In D4Z4-2.5/Dnmt3b^MommeD14^ mice, we measured higher DUX4 transcript levels in the secondary lymphoid organs. Since we had not yet tested DUX4 expression in these tissues in D4Z4-2.5/Smchd1^MommeD1^ mice, we confirmed enhanced DUX4 transcript levels in comparison to D4Z4-2.5 mice (Fig. S2). Previously, we showed that the Smchd1^MommeD1^ variant did not disturb T cell development in the thymus of D4Z4-2.5 mice, although the thymus had a smaller size in mice with significantly enhanced DUX4 transcript levels [18]. Here, we tested whether immune cell populations in the spleen and inguinal lymph nodes of D4Z4-2.5/Smchd1^MommeD1^ and D4Z4-2.5/Dnmt3b^MommeD14^ mice were disturbed by flow cytometry at postnatal day 15. The spleen of D4Z4-2.5/Smchd1^MommeD1^ mice showed a higher number of B cells, the most abundant splenic cell population; however, this was not significant in comparison to D4Z4-2.5 mice. Furthermore, fewer myeloid cells (CD11b^+^) and T cells (CD90^+^) were detected in the spleen of D4Z4-2.5/Smchd1^MommeD1^ mice in comparison to D4Z4-2.5 mice. Within the T cell population, a higher percentage of CD4 T cells were regulatory T cells (Tregs; CD25^+^/Foxp3^+^). In addition, the ratio of CD8 T cells (CD90^+^/CD4^-^/CD8^+^) was increased and fewer CD4 T cells (CD90^+^/CD4^+^/CD8^-^) were detected. Finally, in D4Z4-2.5/Smchd1^MommeD1^ mice more CD8 T cells were identified as memory CD8 T cells (CD44^+^) instead of naïve CD8 T cells (CD44^-^) (Fig. 6a). In contrast, in the spleen of D4Z4-2.5/Dnmt3b^MommeD14^ mice, we only detected a slight decrease in CD4 T cells in comparison to D4Z4-2.5 mice. Other immune cell populations were not affected by the Dnmt3b^MommeD14^ variant (Fig. 6b).

In the inguinal lymph nodes of D4Z4-2.5/Smchd1^MommeD1^ mice, we found similar changes in immune cell populations except from an increased ratio of myeloid cells (Fig. S3A). Again, the immune cell populations in the inguinal lymph nodes of D4Z4-2.5/Dnmt3b^MommeD14^ mice were not altered (Fig. S3B). Importantly, no immune cell defects were found in D4Z4-2.5 mice, Dnmt3b^MommeD14^ mice and Smchd1^MommeD1^ mice. Although both the Smchd1^MommeD1^ and the Dnmt3b^MommeD14^ variants increased DUX4 expression by approximately 5–6 times in the spleen and 8 times in the lymph nodes, the Dnmt3b^MommeD14^ variant did not affect immune cell populations in secondary lymphoid organs. We therefore hypothesized that changes in immune cell subsets in D4Z4-2.5/Smchd1^MommeD1^ mice could be induced by an ongoing inflammation in other tissues. Previously, we identified immune cell infiltrates in the dermis of D4Z4-2.5/Smchd1^MommeD1^ mice that present with upregulated DUX4 expression levels in the skin unlike D4Z4-2.5/Dnmt3b^MommeD14^ mice [18]. To determine whether the skin in the D4Z4-2.5/Dnmt3b^MommeD14^ mice is inflamed as well, we measured different pro-inflammatory cytokines by RT-qPCR. Transcript levels of TNFα, IFNγ, IL-6 and IL-1β were upregulated in the muzzle skin in D4Z4-2.5/Smchd1^MommeD1^ mice in comparison to D4Z4-2.5 mice (Fig. S4A). The transcript levels of the same cytokines were not enhanced in the muzzle skin of D4Z4-2.5/Dnmt3b^MommeD14^ mice (Fig. S4B), indicating the absence of an inflammatory reaction. This may explain why we only found changes in immune cell populations in D4Z4-2.5/Smchd1^MommeD1^ mice and not in D4Z4-2.5/Dnmt3b^MommeD14^ mice.

## Discussion

To study the epigenetic regulation of the D4Z4 repeat array in vivo, the transgenic D4Z4-2.5 mouse model was generated that carries a FSHD1-sized D4Z4 repeat array. This mouse model largely mimics the epigenetic mechanisms to derepress DUX4 as described in FSHD patients [17]. Previously, we showed that cross-breeding D4Z4-2.5 mice with Smchd1^MommeD1^ mice aggravated the phenotype of D4Z4-2.5 mice [18]. In this study, we examined the modifying role of Dnmt3b on DUX4 repression. By cross-breeding hemizygous D4Z4-2.5 mice with heterozygous Dnmt3b^MommeD14^ mice [17, 24], we aimed to recreate the situation in FSHD2 individuals carrying a DNMT3B mutation with the objective to gain more insight into the modifying role of Dnmt3b on DUX4 repression in skeletal muscle and non-muscle tissues.

We previously showed that pups born from cross-breeding hemizygous D4Z4-2.5 mice with heterozygous Smchd1^MommeD1^ mice did not develop a skeletal muscle phenotype at postnatal day 15 even though D4Z4-2.5/Smchd1^MommeD1^ mice showed elevated DUX4 transcript levels in the quadriceps muscle and in myoblast and myotube cultures derived from the EDL muscle. The development of skeletal muscle pathology in the D4Z4-2.5/Smchd1^MommeD1^ mice could not be studied during adulthood as these mice had to be euthanized at a young age [18]. D4Z4-2.5/Dnmt3b^MommeD14^ mice derived from cross-breeding hemizygous D4Z4-2.5 mice with heterozygous Dnmt3b^MommeD14^ mice did not show increased DUX4 expression levels in different skeletal muscles or in myoblast and myotube cultures derived from the EDL muscle. In addition, these mice did not develop skeletal muscle pathology during adulthood making us conclude that skeletal muscles are not sensitive to the Dnmt3b^MommeD14^ variant. Our data nevertheless demonstrates that the transgenic D4Z4-2.5 mouse model can be used to study the role of different epigenetic modifiers at the FSHD locus in vivo. However, it seems that this mouse model will not develop a skeletal muscle phenotype by cross-breeding it with mice carrying variants in epigenetic modifiers of DUX4. To study skeletal muscle pathology in vivo, the FLExDUX4/Acta-C, iDUX4, and TIC-DUX4 mouse models can be used [30–32].

In general, the Smchd1^MommeD1^ variant appeared to have a more potent modifying role on the epigenetic regulation of the D4Z4 repeat array than the Dnmt3b^MommeD14^ variant. D4Z4-2.5/Smchd1^MommeD1^ mice presented with a severe runting phenotype while D4Z4-2.5/Dnmt3b^MommeD14^ mice appeared healthy and reached adulthood (we followed the mice up to 6 months of age) [18]. Furthermore, the Smchd1^MommeD1^ variant more severely impaired the D4Z4 repeat array chromatin structure, and DUX4 transcript levels were increased in more tissues. An explanation for the minor modifying role of the Dnmt3b^MommeD14^ variant could be that this is a hypomorphic variant, and there may be some residual activity despite the predicted structural disruption of the ADD domain [24]. In contrast, the Smchd1^MommeD1^ variant is a nonsense mutation that results in significantly lower *Smchd1* mRNA levels. Several mouse models with variants in *Dnmt3b* have been generated. Mice with a complete loss of the Dnmt3b protein die during embryonic development while mice with homozygous hypomorphic *Dnmt3b* variants show ICF-like phenotypes [33, 34]. Heterozygous Dnmt3b^MommeD14^ mice, which we used for this study, do not present with a phenotype, indicating that this model has a mild *Dnmt3b* variant. However, we believe that the Dnmt3b^MommeD14^ mouse model is a suitable model to represent the variants identified in the two FSHD families as these patients carry missense mutations leading to a single amino acid substitution and are not expected to severely affect the functionality of DNMT3B [21]. In contrast, SMCHD1 variants found in FSHD families include nonsense and insertion-deletion variants. As the SMCHD1 protein forms dimers, SMCHD1 variants that preserve the open reading frame most likely exert a dominant negative effect [14, 15, 22, 35]. Another explanation for the milder effect of the Dnmt3b^MommeD14^ variant at the D4Z4 repeat array is that SMCHD1 might have a more potent role in epigenetic repression of the D4Z4 repeat array than DNMT3B. Many FSHD2 patients have been identified with mutations in SMCHD1, in contrast to DNMT3B, where mutations thus far have only been identified in two families that carry permissive 4qA alleles with only 9 or 13 D4Z4 units. Therefore, our findings are consistent with the number of FSHD2 patients identified with mutations in either SMCHD1 or DNMT3B [13, 21, 35].

In this study, we found increased DUX4 transcript levels in the spleen and inguinal lymph nodes of both D4Z4-2.5/Smchd1^MommeD1^ and D4Z4-2.5/Dnmt3b^MommeD14^ mice. In D4Z4-2.5/Dnmt3b^MommeD14^ mice, increased DUX4 levels in comparison to D4Z4-2.5 mice remained restricted to the secondary lymphoid organs. It seems that these immune tissues are more sensitive to epigenetic modifiers of the D4Z4 repeat array. As Dnmt3b is not enriched at the D4Z4 repeat in the spleen, the Dnmt3b^MommeD14^ variant likely causes a failure of DNA methylation establishment at the D4Z4 repeat during early development. Early embryonic experiments, for example by differentiating mESCs into different somatic cell types, will need to be performed to establish why these immune tissues are more sensitive.

To establish the consequences of enhanced DUX4 expression in the spleen and inguinal lymph nodes, we characterized different immune cell populations from these tissues of pups derived from cross-breeding hemizygous D4Z4-2.5 mice with either heterozygous Smchd1^MommeD1^ mice or heterozygous Dnmt3b^MommeD14^ mice. Although the variants in *Smchd1* and *Dnmt3b* both increased DUX4 expression in the spleen and lymph nodes at a similar level, only immune cell populations in the D4Z4-2.5/Smchd1^MommeD1^ mice were affected. The most important findings in the spleen were the reduction of T cells and myeloid cells and an increase in B cells. Within the T cell population, the percentage of regulatory T cells and CD8 cell was increased and the CD8 T cells showed a shift towards memory T cells instead of naïve T cells. These changes might indicate a systemic infection induced by a higher DUX4 expression; however, histopathological examination only revealed inflammatory cell infiltrations in the dermis of the D4Z4-2.5/Smchd1^MommeD1^ mice [18]. The lack of changes in immune cell populations in the secondary lymphoid organs of D4Z4-2.5/Dnmt3b^MommeD14^ mice might indicate the absence of an inflammatory reaction in other organs. These results highlight that D4Z4-2.5/Smchd1^MommeD1^ mice overall show a more severe phenotype than D4Z4-2.5/Dnmt3b^MommeD14^ mice. Up to now, the role of the immune system in FSHD patients has been understudied. The mis-expression of DUX4 in skeletal muscles may activate the immune system. Pro-inflammatory genes and immune cells are detected in FSHD muscle biopsies [36, 37]; however, it is unknown if the immune system is activated by the apoptosis of skeletal muscle cells, mis-expression of DUX4, or both. Also, it is unknown whether DUX4 is expressed in immune tissues or other non-muscle tissues of FSHD patients; however, no lympho-hematopoietic defects have been described in patients. DUX4 expression has been reported in the thymus and epidermis of non-affected individuals [38, 39], suggesting a potential role for DUX4 in somatic tissues. Our observation is that the D4Z4 transgene array in the immune system is particularly sensitive to epigenetic modifiers, therefore the D4Z4-2.5 mouse model may be used to further study the role of DUX4 in the immune system.

## Conclusions

In this study, we determined that the Dnmt3b^MommeD14^ variant does not induce a skeletal muscle pathology nor does it increase the extremely low DUX4 transcript levels in skeletal muscles of transgenic D4Z4-2.5 mice. In secondary lymphoid organs, DNA methylation at the D4Z4 repeat array was reduced and DUX4 transcript levels were enhanced in comparison to D4Z4-2.5 mice. Next, we showed that immune cell subsets were disturbed in secondary lymphoid organs of D4Z4-2.5/Smchd1^MommeD1^ mice but not in D4Z4-2.5/Dnmt3b^MommeD14^ mice. We conclude that *Smchd1* may have a more potent role in DUX4 derepression than *Dnmt3b* in transgenic FSHD mice, which corresponds with the number of FSHD patients identified with either variants in SMCHD1 or DNMT3B. Additionally, the D4Z4-2.5/Smchd1^MommeD1^ mouse model may be used to study the role of DUX4 in the immune system in relation to FSHD pathology.

## Supplementary information

**Additional file 1: Figure S1.** Wfdc3 expression (DUX4 target gene) in wild-type, Dnmt3b^MommeD14^, D4Z4-2.5, and D4Z4-2.5/Dnmt3b^MommeD14^ mice (postnatal day 15). In skeletal muscles (**A**), *Wfdc3* expression is barely changed in different genotypes as determined by RT-qPCR while in secondary lymphoid organs (**B**), *Wfdc3* is mostly enhanced in D4Z4-2.5/Dnmt3b^MommeD14^ mice. Each dot represents one mouse and the error bars represent the standard error of the mean (SEM) from biological replicates. Statistical analysis was performed with a Kruskal–Wallis test. *P<0.05; **P<0.01. Other results were not significant. WT= wild type; 2.5 = D4Z4-2.5; MD14 = Dnmt3b^MommeD14^.

**Additional file 2: Figure S2.** The Smchd1^MommeD1^ variant affects DUX4 expression in secondary lymphoid organs of D4Z4-2.5 mice. DUX4 transcript levels were enhanced in the inguinal lymph nodes and spleens of D4Z4-2.5/Smchd1^MommeD1^ mice (postnatal day 15) as measured by RT-qPCR. Each dot represents one mouse and the error bars denote the SEM from the biological replicates. Statistical analysis was performed with a Student’s t-test. **P<0.01; ***P<0.001. 2.5 = D4Z4-2.5; MD1 = Smchd1^MommeD1^.

**Additional file 3: Figure S3.**. Immune cell populations in the inguinal lymph nodes of D4Z4-2.5/Smchd1^MommeD1^ showed different ratios, while all immune cell populations in the D4Z4-2.5/Dnmt3b^MommeD14^ were unaffected. Immune cells from the inguinal lymph nodes from pups (postnatal day 15) derived from cross-breeding hemizygous D4Z4-2.5 mice with heterozygous Smchd1^MommeD1^ mice (**A**) or from cross-breeding hemizygous D4Z4-2.5 mice with heterozygous Dnmt3b^MommeD14^ mice (**B**) were stained with different antibodies and detected using flow cytometry. The average percentage of immune cells found in wild-type mice in each litter was set to 1 and ratios were calculated per experiment. Each dot represents one mouse. Statistical analysis was performed using one-way ANOVA. *P<0.05; **P<0.01; ***P<0.001. Other results were not significant. 2.5 = D4Z4-2.5; MD1 = Smchd1^MommeD1^; MD14 = Dnmt3b^MommeD14^.

**Additional file 4: Figure S4.** Expression of pro-inflammatory cytokines in the muzzle skin is enhanced in D4Z4-2.5/Smchd1^MommeD1^ mice. Transcript levels of cytokines TNFα, IFNγ, IL-6 and IL-1β in the muzzle skin as measured by RT-qPCR were upregulated in D4Z4-2.5/Smchd1^MommeD1^ mice **(A)** but not in D4Z4-2.5/Dnmt3b^MommeD14^ mice (postnatal day 15) **(B)**. Each dot represents one mouse and the error bars denote the SEM of five biological replicates. For the D4Z4-2.5/Smchd1^MommeD1^ mice, statistical analysis was performed with a Student’s t-test (IL-6 and IL-1β) or a Mann–Whitney U test (TNFα, IFNγ). Statistical analysis of cytokine expression in the D4Z4-2.5/Dnmt3b^MommeD14^ mice was performed with a Student’s t-test (TNFα, IFNγ) or a Mann–Whitney U test (IL-6 and IL-1β). **P<0.01; ***P<0.001. n.s. = not significant. 2.5 = D4Z4-2.5; MD1 = Smchd1^MommeD1^; MD14 = Dnmt3b^MommeD14^.

## Data Availability

Most data that was generated for this study is included in the manuscript and the supplementary information. The corresponding author can be contacted if additional data is required.

## Abbreviations

ADD: ATRX-Dnmt3-Dnmt3L
DNMT3B: DNA methyltransferase 3B
EDL: Extensor digitorum longus
FSHD: Facioscapulohumeral muscular dystrophy
ICF: Immunodeficiency, Centromeric instability, and Facial anomalies
mESCs: Mouse embryonic stem cells
Tregs: Regulatory T cells
SMCHD1: Structural maintenance of chromosomes flexible hinge domain-containing 1
