# Supplementary figures and images for: Dnmt3b regulates DUX4 expression in a tissue-dependent manner in transgenic D4Z4 mice
Source: Skelet Muscle. 2020 Oct 1;10:27. doi: 10.1186/s13395-020-00247-0 (PMC7528343; doi:10.1186/s13395-020-00247-0)

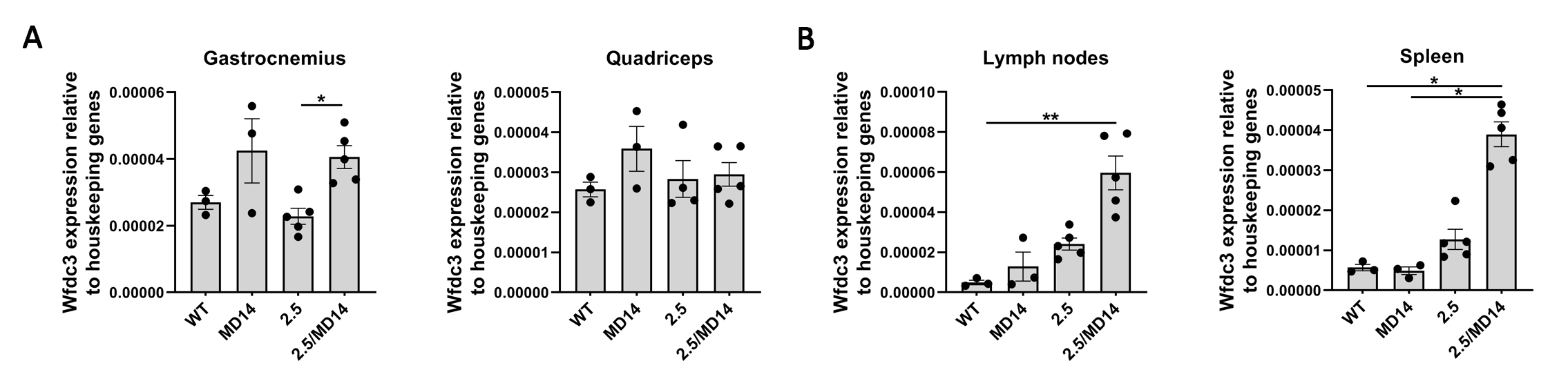

Supplement: Supplementary file 1 — Additional file 1: Figure S1. Wfdc3 expression (DUX4 target gene) in wild-type, Dnmt3bMommeD14, D4Z4-2.5, and D4Z4-2.5/Dnmt3bMommeD14 mice (postnatal day 15). In skeletal muscles (A), Wfdc3 expression is barely changed in different genotypes as determined by RT-qPCR while in secondary lymphoid organs (B), Wfdc3 is mostly enhanced in D4Z4-2.5/Dnmt3bMommeD14 mice. Each dot represents one mouse and the error bars represent the standard error of the mean (SEM) from biological replicates. Statistical analysis was performed with a Kruskal–Wallis test. *P<0.05; **P<0.01. Other results were not significant. WT= wild type; 2.5 = D4Z4-2.5; MD14 = Dnmt3bMommeD14. [file 13395_2020_247_MOESM1_ESM.tif]

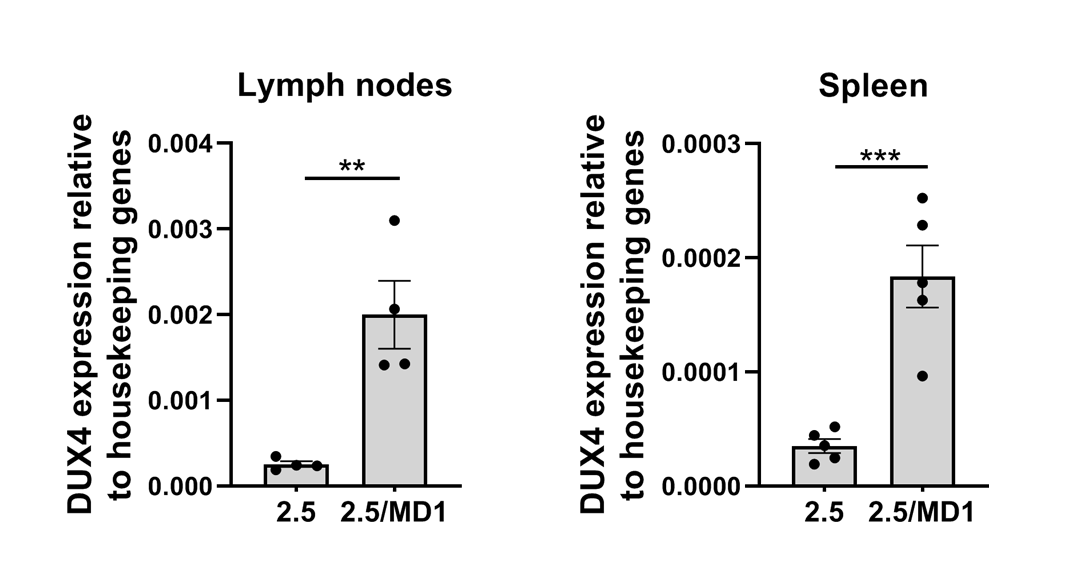

Supplement: Supplementary file 2 — Additional file 2: Figure S2. The Smchd1MommeD1 variant affects DUX4 expression in secondary lymphoid organs of D4Z4-2.5 mice. DUX4 transcript levels were enhanced in the inguinal lymph nodes and spleens of D4Z4-2.5/Smchd1MommeD1 mice (postnatal day 15) as measured by RT-qPCR. Each dot represents one mouse and the error bars denote the SEM from the biological replicates. Statistical analysis was performed with a Student’s t-test. **P<0.01; ***P<0.001. 2.5 = D4Z4-2.5; MD1 = Smchd1MommeD1. [file 13395_2020_247_MOESM2_ESM.tif]

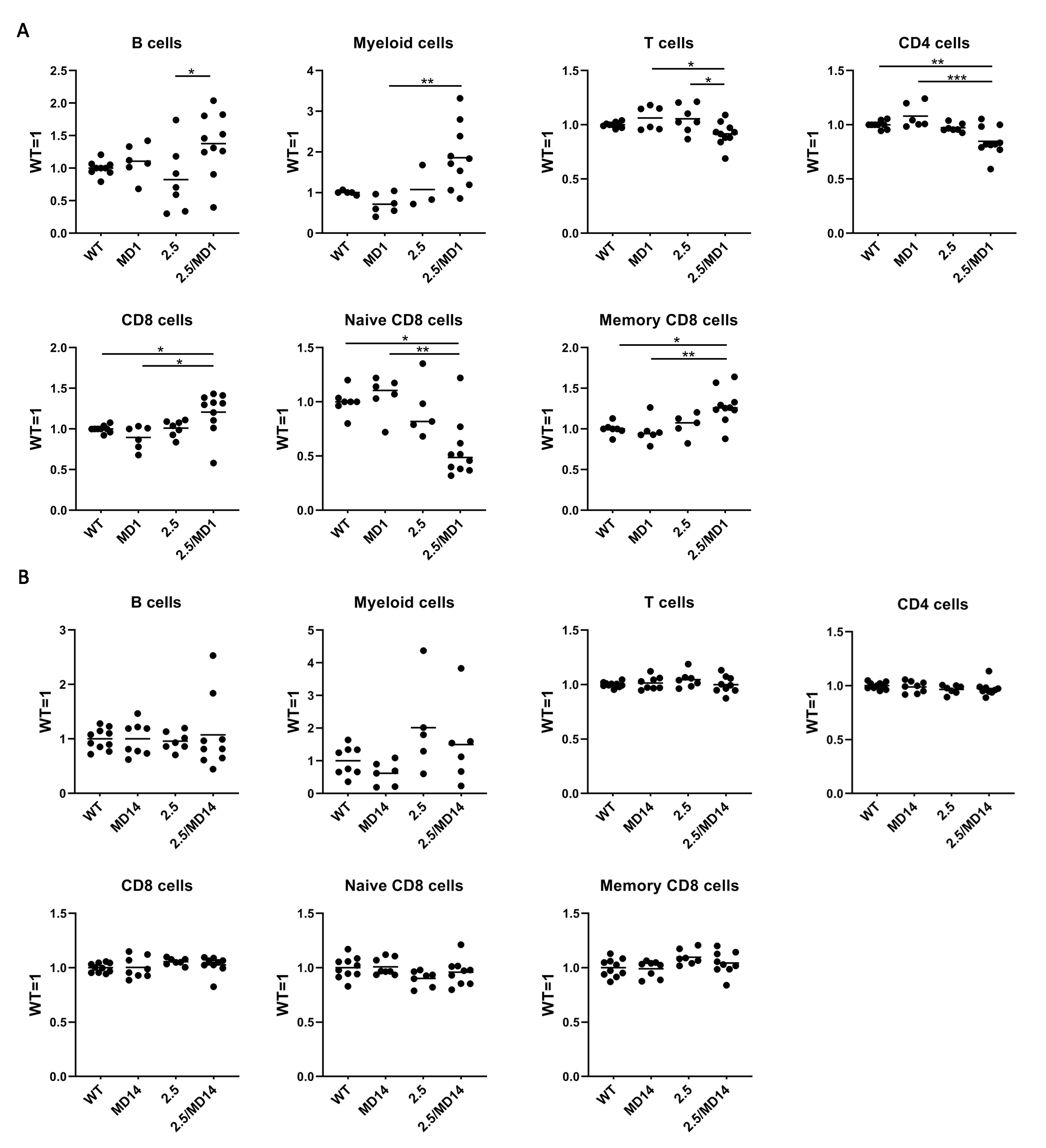

Supplement: Supplementary file 3 — Additional file 3: Figure S3.. Immune cell populations in the inguinal lymph nodes of D4Z4-2.5/Smchd1MommeD1 showed different ratios, while all immune cell populations in the D4Z4-2.5/Dnmt3bMommeD14 were unaffected. Immune cells from the inguinal lymph nodes from pups (postnatal day 15) derived from cross-breeding hemizygous D4Z4-2.5 mice with heterozygous Smchd1MommeD1 mice (A) or from cross-breeding hemizygous D4Z4-2.5 mice with heterozygous Dnmt3bMommeD14 mice (B) were stained with different antibodies and detected using flow cytometry. The average percentage of immune cells found in wild-type mice in each litter was set to 1 and ratios were calculated per experiment. Each dot represents one mouse. Statistical analysis was performed using one-way ANOVA. *P<0.05; **P<0.01; ***P<0.001. Other results were not significant. 2.5 = D4Z4-2.5; MD1 = Smchd1MommeD1; MD14 = Dnmt3bMommeD14. [file 13395_2020_247_MOESM3_ESM.tif]

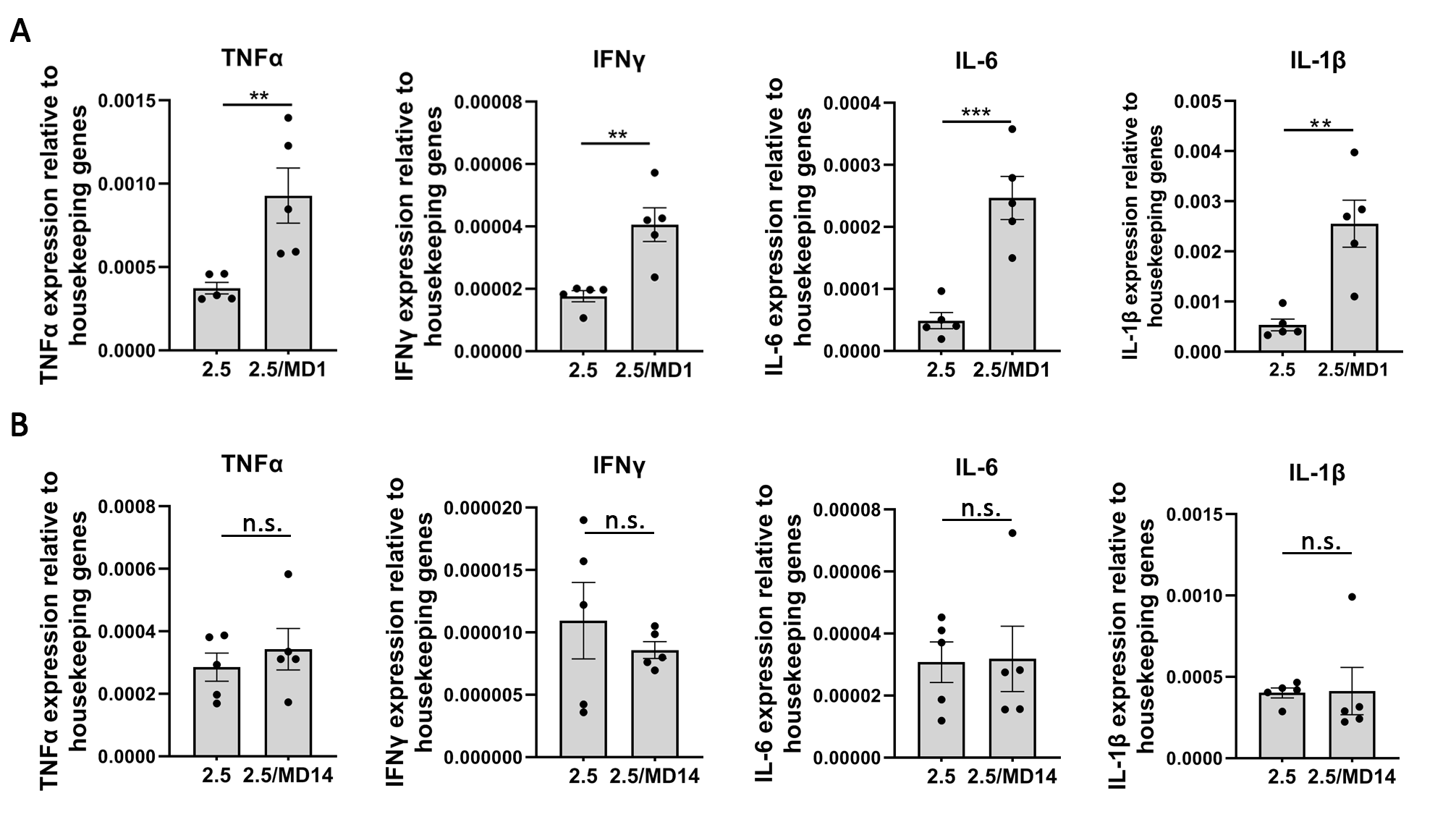

Supplement: Supplementary file 4 — Additional file 4: Figure S4. Expression of pro-inflammatory cytokines in the muzzle skin is enhanced in D4Z4-2.5/Smchd1MommeD1 mice. Transcript levels of cytokines TNFα, IFNγ, IL-6 and IL-1β in the muzzle skin as measured by RT-qPCR were upregulated in D4Z4-2.5/Smchd1MommeD1 mice (A) but not in D4Z4-2.5/Dnmt3bMommeD14 mice (postnatal day 15) (B). Each dot represents one mouse and the error bars denote the SEM of five biological replicates. For the D4Z4-2.5/Smchd1MommeD1 mice, statistical analysis was performed with a Student’s t-test (IL-6 and IL-1β) or a Mann–Whitney U test (TNFα, IFNγ). Statistical analysis of cytokine expression in the D4Z4-2.5/Dnmt3bMommeD14 mice was performed with a Student’s t-test (TNFα, IFNγ) or a Mann–Whitney U test (IL-6 and IL-1β). **P<0.01; ***P<0.001. n.s. = not significant. 2.5 = D4Z4-2.5; MD1 = Smchd1MommeD1; MD14 = Dnmt3bMommeD14. [file 13395_2020_247_MOESM4_ESM.tif]
